# A robust flow cytometry-based biomass monitoring tool enables rapid at-line characterization of *S. cerevisiae* physiology during continuous bioprocessing of spent sulfite liquor

**DOI:** 10.1007/s00216-020-02423-z

**Published:** 2020-02-07

**Authors:** Charlotte Anne Vees, Lukas Veiter, Fritz Sax, Christoph Herwig, Stefan Pflügl

**Affiliations:** 1 Institute of Chemical, Environmental and Bioscience Engineering, Research Area Biochemical Engineering, Technische Universität Wien, Gumpendorfer Straße 1a, 1060 Vienna, Austria; 2 Christian Doppler Laboratory for Mechanistic and Physiological Methods for Improved Bioprocesses, TU Wien, Gumpendorfer Straße 1a, 1060 Vienna, Austria; 3 Competence Center CHASE GmbH, Altenbergerstraße 69, 4040 Linz, Austria

**Keywords:** Sustainable bioprocess solution, Yeast morphology, Viable/non-viable biomass populations, Particle background, Complex medium, Continuous bioprocessing with cell retention

## Abstract

Assessment of viable biomass is challenging in bioprocesses involving complex media with distinct biomass and media particle populations. Biomass monitoring in these circumstances usually requires elaborate offline methods or sophisticated inline sensors. Reliable monitoring tools in an at-line capacity represent a promising alternative but are still scarce to date. In this study, a flow cytometry-based method for biomass monitoring in spent sulfite liquor medium as feedstock for second generation bioethanol production with yeast was developed. The method is capable of (i) yeast cell quantification against medium background, (ii) determination of yeast viability, and (iii) assessment of yeast physiology though morphological analysis of the budding division process. Thus, enhanced insight into physiology and morphology is provided which is not accessible through common online and offline biomass monitoring methods. To demonstrate the capabilities of this method, firstly, a continuous ethanol fermentation process of *Saccharomyces cerevisiae* with filtered and unfiltered spent sulfite liquor media was analyzed. Subsequently, at-line process monitoring of viability in a retentostat cultivation was conducted. The obtained information was used for a simple control based on addition of essential nutrients in relation to viability. Thereby, inter-dependencies between nutrient supply, physiology, and specific ethanol productivity that are essential for process design could be illuminated.

**Figure:**
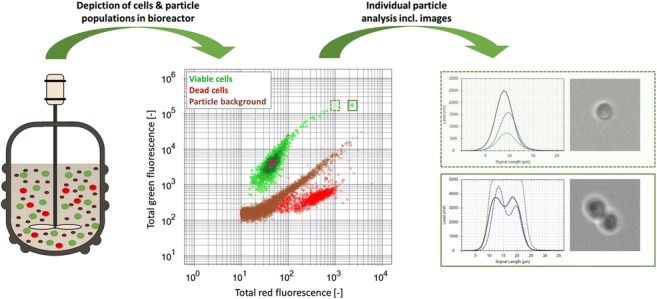
Graphical abstract

Graphical abstract

## Introduction

In recent years, spent sulfite liquor (SSL) has attracted attention as an attractive feedstock for second generation bioethanol production using genetically engineered baker’s yeast [1]. As an abundant and cheap by-product of the sulfite cooking process of wood for pulp and paper production, spent sulfite liquor contains high amounts of a variety of different hexose and pentose sugars [2–5]. During pulping, lignocellulosic material is hydrolyzed into a solid cellulose fraction used for paper and viscose production and a liquid fraction containing mainly sugar monomers from hemicellulose [1, 2, 6]. Sugars are directly available and costly pretreatment can be avoided, which makes the biorefinery of spent sulfite liquor to ethanol economically feasible [4]. Nevertheless, hydrolysis also leads to accumulation of lignosulfonates, sulfate, and a variety of inhibitory break down products, like acetic acid, furfural, and hydroxymethylfurfural (HMF). Lignosulfonates mainly contribute to a high solid particle content of the spent sulfite liquor [7, 8]. HMF and furfural have a still not fully explored inhibitory effect on yeast growth and ethanol productivity [9]. While acetic acid can be co-utilized as an additional carbon source in addition to sugars by commonly used biotechnological production hosts *Saccharomyces cerevisiae* and *Escherichia coli* [10–12], it has a strong influence on the cytosolic pH and can negatively influence viability [2, 13]. Beside those challenges, the biorefinery of spent sulfite liquor provides the opportunity to produce sustainable biofuels by valorization of the waste stream. It does not compete with food production—like first-generation feedstocks—and applies zero-waste conversion technologies, a key component in future circular economy technologies [4, 14, 15].

For an economic and ecological bioprocessing of the continuously generated large quantities of spent sulfite liquor, bioprocessing via continuous fermentation of is essential. It leads to an increased productivity and high time-space yields in ethanol production. The inhibiting conditions in spent sulfite liquor processes lead to deteriorating growth rate, viability, and fermentation performance [16]. Consequently, maintaining steady cell viability and high biomass concentration is the main challenge in generating a stable and productive process. A promising strategy to meet these demands is to uncouple growth from product formation by cell retention in a retentostat. Previous retentostat experiments showed an accumulation of solid particles in the cell retention process despite pre-filtration of spent sulfite liquor (data not shown). The increased particle content leads to inaccurate biomass measurements which impedes determination of essential variables for process understanding such as growth rates, substrate uptake rates, and biomass yield [17]. Consequently, the in situ measurement of the viable biomass and cell count is essential for systematic optimization of cultivation parameters in the continuous cell retention process.

So far, determination of cell viability in spent sulfite liquor has been achieved mainly through alkaline methylene blue method and by counting the colony forming units on agar plates [1, 16, 18], which is time consuming, negatively affected by high particle backgrounds and cannot depict the physiology of different biomass populations. In an industrial setting, physical techniques capable of real time measurement are preferred [19]. Common methods for in situ measurement of viable biomass include dielectric spectroscopy, infrared spectroscopy and fluorescence spectroscopy, NIR spectroscopy, and Raman spectroscopy as well as microscopy combined with image analysis [19–23]. However, inline sensors are prone to high measurement noise and require chemometric knowledge to establish meaningful measurement techniques or display limitations in other fields [20]. For instance, turbidity probes are not feasible in combination with high particle background in complex media [24]; commercial dielectric spectroscopy probes can differentiate between viable cells and other solid particles, but cannot quantify the amount of dead cells and particle background. These techniques have also exhibited polarization problems when medium conductivity is high [20, 21]. In particle-free medium, near-infrared spectroscopy (NIR) and Raman spectroscopy are powerful tools for a fast and non-invasive determination of substrate concentration, product formation, and viable biomass concentration [23, 25–27]. In complex medium containing a high particle load—like lignocellulose hydrolysate or spent sulfite liquor—it is not possible to differentiate between viable cells and solid medium particles with NIR and Raman [25, 28]. According to Ewanick et al. [29], lignocellulose hydrolysate medium pretreated with filtration certainly requires extensive modeling to reduce baseline shifts and fluctuating spectral background.

Flow cytometry in combination with fluorescent viability staining [30, 31] is a promising alternative when dealing with complex media containing particles and emulsified liquids. Thereby, the entire particle population is depicted in a quantitative way [32], including viable and non-viable biomass against media background. Furthermore, morphological assessment of biomass or analysis of media particles is possible [33]. In recent years, efforts to use flow cytometry in online mode have been successfully undertaken [34–37]. In this context, automated sample treatment involving dilution, fluorescent staining, and incubation is still a considerable bottleneck; however, for this purpose, automated sampling and sample processing systems have been developed recently [38].

In this study, a flow cytometry-based method to analyze yeast cells in complex media containing spent sulfite liquor with high particle background was developed. The method is capable of (i) yeast cell quantification against medium background, (ii) determination of yeast viability, and (iii) assessment of yeast physiology though morphological analysis of the budding division process. The method was successfully employed as a monitoring tool in fermentation processes of *S. cerevisiae* in spent sulfite liquor: first, the method was verified in chemostat processes at different biomass concentrations, and subsequently physiology and morphology under cell retention conditions were assessed.

## Materials and methods

### Pre-culture preparation

Baker’s yeast stored at − 80 °C glycerol stocks (1.5 mL) was directly inoculated into 100 mL YPD medium (10 g L^−1^ yeast extract, 20 g L^−1^ peptone, and 20 g L^−1^ glucose; pH 6.5) in 500 mL shake flasks and incubated for 18 h at 32 °C and 200 rpm. The pre-culture was then centrifuged (4000 rpm, 10 min, 20 °C), washed once with 100 mL 0.9% (w/v) NaCl, and resuspended in 10 mL of the respective batch medium.

### Spent sulfite liquor medium

Spent sulfite liquor with a dry matter content of 30–32% (w/v) from an industrial source was used for all experiments in this study. Spent sulfite liquor served as the carbon source, containing approximately 12% (w/v) hexose and pentose sugars. In addition, per liter medium 15 mL phosphate stock solution (21.7 g L^−1^ K_2_HPO_4_, 182.3 g L^−1^ KH_2_PO_4_) and 5 mL L^−1^ of a urea stock solution (400 g L^−1^ urea) were aseptically added to unfiltered or filtered spent sulfite liquor and the pH was adjusted to 5.0 or 5.5 with Mg (OH)_2_.

For continuous cultivations using cell retention, filtration of the medium was required to avoid blocking the cell retention membrane by solid spent sulfite liquor particles. To reduce major impurities, a pre-filtration step through a commercial cloth strainer and fine filtration via continuous crossflow filtration using a Pall PSP-113 polyolefin hollow-fiber membrane (Pall Corporation, New York, USA) were carried out.

### Cultivations in bioreactors

The chemostat process was done in four parallel 3-L DASGIP® Benchtop Bioreactors (Eppendorf AG, Hamburg, Germany), while the fermentation with cell retention was carried out in a 1.5-L stirred tank glass bioreactor (Applikon Biotechnology BV, Delft, Netherlands). All reactors had a working volume of 1 L.

Cultivations were started at an OD of 0.5 (chemostats) or 1.0 (retentostat) by adding an appropriate volume of pre-culture to the reactor. For the batch phase, the yeast was cultivated in YPD medium (chemostat) or SSL medium (retentostat). Upon depletion of the carbon source, cultivations were transferred into continuous mode by feeding minimal SSL medium with a constant dilution rate of 0.02 h^−1^ in chemostat and 0.07 h^−1^ in retentostat, which complies with a feed rate of 20 mL h^−1^ and 70 mL h^−1^ respective. The feeding of medium with either unfiltered or filtered spent sulfite liquor in chemostat was carried out in duplicate.

During the batch phases, aerobic conditions were maintained via agitation at 500 rpm (chemostat) or 800 rpm (retentostat) and aeration with air at 1 vvm adjusted by the mass flow controller (Brooks Instrument, Dresden, Germany). The dissolved oxygen was monitored by a VisiFerm DO 225 probe (Hamilton, Reno/NV, USA) or VisiFerm DO 120 probe (Hamilton, Reno/NV, USA), in chemostat or retentostat respective. At the transition to the chemostat phase and the retentostat phase, the agitation was set to 350 rpm (chemostat) or held at 800 rpm (retentostat). For anaerobic conditions in the entire chemostat phase and the respective anaerobic retentostat phases, the gas supply was switched to 0.07 vvm nitrogen. Reactor off-gas was analyzed using a DASGIP GA4 gas sensor module (Eppendorf AG, Hamburg, Germany) in the chemostat reactors and a CO_2_ gas sensor module (BlueSens gas sensor GmbH, Hamburg, Germany) in the retentostat. pH was monitored by a pH electrode (Mettler-Toledo GmbH, Giessen, Germany) and controlled at 5.5 during batch and 5.0 during continuous cultivation phases by addition of 2 M KOH. In both processes, the temperature was constantly set at 32 °C.

The full cell retention in the retentostat process was realized by continuously pumping the whole reactor content through a loop including a Pall PSP-113 polyolefin hollow fiber membrane (Pall Corporation, New York, USA). The harvesting was conducted by removing cell-free permeate through the hollow fiber membrane, while the retained cell broth was fed back into the reactor. The harvest rate was adjusted to maintain a constant filling volume of the reactor, realized by a dip tube in the chemostat, and monitored by a DASGIP® level sensor (Eppendorf AG, Hamburg, Germany) in the retentostat.

For the supplementation of a nutrient-pulse into the reactor, a solution of 10 g peptone and 5 g yeast extract in 50 mL demineralized water was prepared.

### Flow cytometry

Samples from cultivations were diluted 1:10 into phosphate-buffered saline (50 g L^−1^ of 2.65 g L^−1^ CaCl_2_ solution, 0.2 g L^−1^ KCl, 0.2 g L^−1^ KH_2_PO_4_, 0.1 g L^−1^ MgCl · 6 H_2_O, 8 g L^−1^ NaCl and 0.764 g L^−1^ Na_2_HPO_4_ x 2 H_2_O) and stained with propidium iodide (Sigma-Aldrich, St. Louis, MO, USA; 20 mM stock dissolved in DMSO ≥ 99.9%, diluted with phosphate-buffered saline to a final concentration of 20 μM). After incubating 1 min, the sample was further stained with fluorescein diacetate (Sigma-Aldrich, St. Louis, MO, USA; stock solution of 5 g L^−1^ dissolved in acetone ≥ 99.9% to a final concentration of 5 mg L^−1^). After an incubation time of 10 min, the sample was further diluted (1:100 in the same buffer) for flow cytometric analysis.

For calibration of the method, a yeast pre-culture was centrifuged (4000 rpm, 10 min, 20 °C) and dissolved with PBS buffer to reach an optical density of 1. To study various viability stages, one half of the solution was subjected to microwave treatment for 30 s at 940 W in a microwave oven. Subsequently, mixtures of viable and dead cells were prepared in several ratios to identify viable and non-viable populations. For identification of background noise in the medium, either raw or filtered SSL medium was added to the cell mixtures in pure buffer.

Table 1 shows the mixtures of viable and dead cell suspensions measured either in PBS, unfiltered, or filtered SSL medium. In addition, cell and spent sulfite liquor concentrations were varied to test the effect on measurement accuracy.

**Table 1:** Overview of the mixtures of viable (V) and dead (D) cell solutions, measured in phosphate-buffered saline (PBS), unfiltered SSL medium, or filtered SSL medium. Mixing ratios were combined with a variation of the optical density (OD) of the cells and the variation of the added spent sulfite liquor

V/D (%)	100/0	80/20	60/40	50/50	40/60	20/80	0/100
PBS	1 OD	1 OD	0.5 OD	1 OD	0.5 OD	1 OD	1 OD
			1 OD		1 OD		
			2 OD		2 OD		
Unfiltered SSL medium	1 OD	1 OD	0.5 OD	1 OD each with ½ SSL, 1 SSL, 2 SSL	0.5 OD	1 OD	1 OD
			1 OD		1 OD		
			2 OD		2 OD		
Filtered SSL medium	1 OD	1 OD	0.5 OD	1 OD each with ½ SSL, 1 SSL, 2 SSL	0.5 OD	1 OD	1 OD
			1 OD		1 OD		
			2 OD		2 OD		

A CytoSense flow cytometer (CytoBuoy, Woerden, Netherlands) was used for all measurements as described previously [33, 39]. Data analysis was performed using the software CytoClus4 (CytoBuoy, Woerden, Netherlands).

### Calculation of expected ratio of viable and dead cells

The expected ratio of viable and dead cells was calculated via Eqs. 1–4. This calculation approach will be discussed in the “Development of flow cytometry-based method” section.

1 $$ {N}_{\exp .,\kern0.5em \mathrm{viable}}=\left({N}_{\mathrm{FDA},\mathrm{V}100\%\kern0.5em }\times \frac{P_{\mathrm{viable}}}{100}\ \right)+\left(\ {N}_{\mathrm{FDA},\mathrm{D}100\%}\times \frac{P_{\mathrm{dead}}\ }{100}\right) $$

2 $$ {N}_{\exp .,\mathrm{dead}}=\left({N}_{\mathrm{PI},\mathrm{V}100\%\kern0.5em }\times \frac{P_{\mathrm{viable}}}{100}\ \left[\%\right]\right)+\left(\ {N}_{\mathrm{PI},\mathrm{D}100\%}\times \frac{P_{\mathrm{dead}}}{100}\ \left[\%\right]\right) $$

3 $$ {\mathrm{Ratio}}_{\mathrm{V},\kern0.5em \exp .}=\frac{\ {N}_{\exp .,\kern0.5em \mathrm{viable}}}{\ {N}_{\exp .,\kern0.5em \mathrm{viable}}+{N}_{\exp .,\kern0.5em \mathrm{dead}}}\times 100 $$

4 $$ {\mathrm{Ratio}}_{\mathrm{D},\kern0.5em \exp .}=\frac{\ {N}_{\exp .,\kern0.5em \mathrm{dead}}}{\ {N}_{\exp .,\kern0.5em \mathrm{viable}}+{N}_{\exp .,\kern0.5em \mathrm{dead}}}\times 100 $$

*N*_exp., viable_: Expected cell count, viable (N/mL)
*N*_exp., dead_: Expected cell count, dead (N/mL)
*N*_FDA, V100%_: Cell count FDA, V100% (N/mL)
*N*_FDA, D100%_: Cell count FDA, D100% (N/mL)
*P*_viable_: Percentage of viable cell solution (%)
*P*_dead_: Percentage of dead cell solution (%)
Ratio_V, exp._: Percentage of viable cells in suspension (%)
Ratio_D, exp._: Percentage of dead cells in suspension (%)

Equations 1–4 describe the calculation of expected ratio of viable (V) and dead (D) cells from expected cell counts.

### Optical density and biomass determination

The optical density was measured in triplicates at a wavelength of 600 nm with a Spectronic 20 Genesys spectrophotometer (Thermo Scientific, Waltham, MA, USA).

The biomass was determined gravimetrically in triplicates. For this purpose, 2 mL culture broth was centrifuged (4500 rpm, 10 min, 4 °C), washed with 4 mL deionized water, and dried in pre-weighed glass tubes for at least 24 h at 105 °C.

### HPLC analysis

Substrate and metabolite concentrations in the culture broth were measured as described previously by Erian et al. [40], using an Ultimate 3000 system (Thermo Scientific, Waltham/MA, USA) using an Aminex HPX-87H column (300 × 7.8 mm, Bio-Rad, Hercules, CA, USA).

## Results

Initial method development was focused on identifying viable and non-viable biomass in various complex media backgrounds featuring spent sulfite liquor. Subsequently, the applicability of the method was tested (i) in a chemostat process with different biomass concentrations and particle backgrounds and (ii) as a process monitoring tool for cell physiology in a retentostat ethanol production process.

### Development of flow cytometry-based method

Method calibration was performed using various biomass concentrations in different viability stages and media compositions. By using flow cytometry in combination with fluorescent staining, a false-positive detection of media particles as biomass could be avoided. For this purpose, we employed two types of fluorescent dyes: (a) fluorescein diacetate (FDA) resulting in green fluorescence through esterase activity [41] to detect metabolic activity of viable biomass and (b) propidium iodide (PI) resulting in red fluorescence as a result of DNA intercalation in cells with compromised membranes [42].

**Fig. 1:**
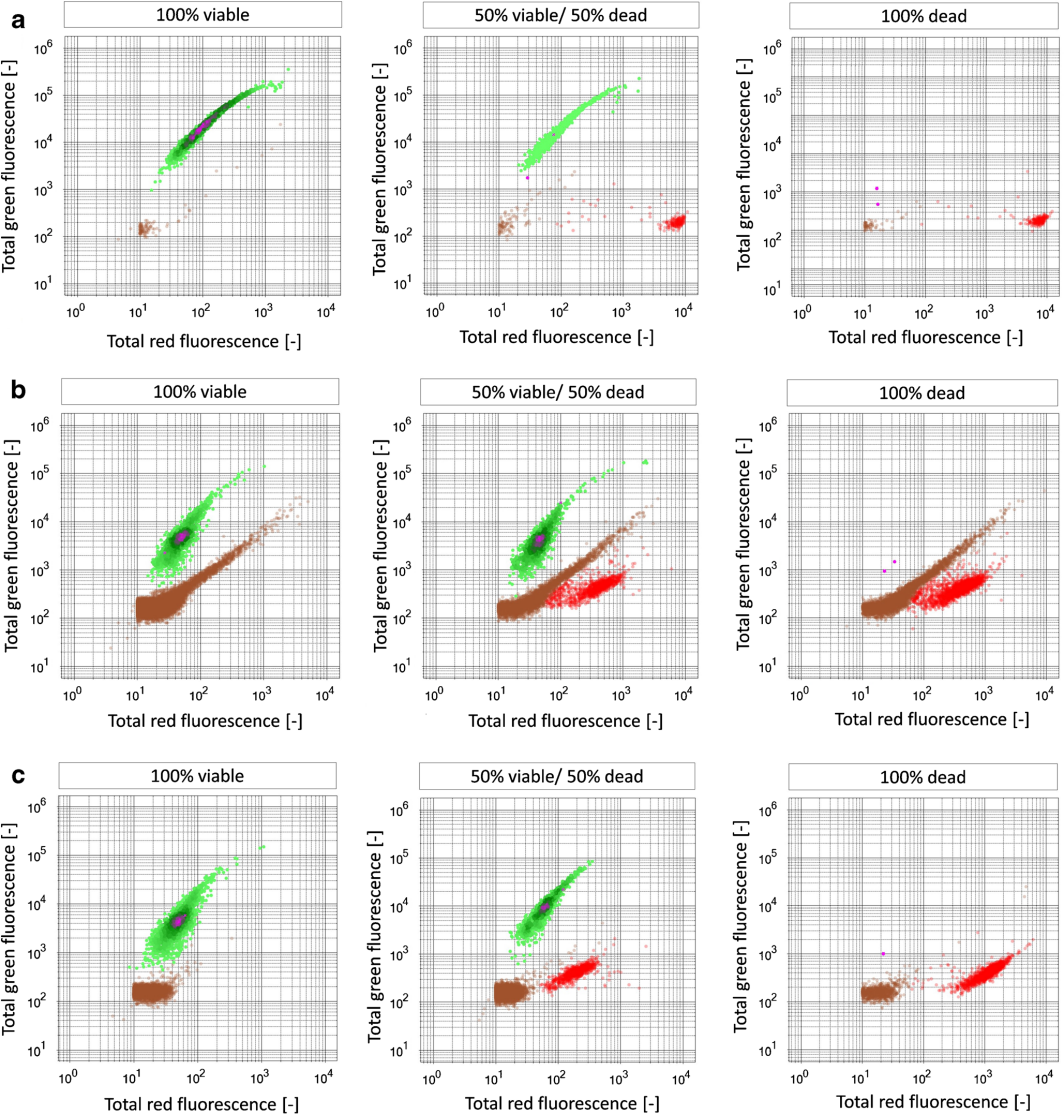
Flow cytometry scatter plots depicting identification of viable cells (green) and dead cells (red) against media background (brown). Columns from left to right: ratio of viable cells vs. dead cells derived from microwave treatment, 100% viable cells (left), 50% viable vs. 50% dead cells (middle), 100% dead cells (right). Rows from top to bottom: **a** cells in particle-free buffer, **b** SSL medium, and **c** filtered SSL medium

For method development, defined volumetric mixtures of viable and dead cells in different media backgrounds were measured. Figure 1 provides an overview of the identified clusters against different media backgrounds. Based on initial measurements of medium (i) with or without spent sulfite liquor and (ii) with and without cells, a distinction of yeast cells from media background was possible (see Fig. 1, middle column). Scatter plots of red and green total fluorescence signals clearly display three clusters: viable cells, dead cells, and media background (see Fig. 1b, center row). At high SSL particle concentrations (see Fig. 1b, c), deviations in red fluorescence caused by particle interaction with PI could be observed. Consequently, biomass identification was not only based on fluorescence but also on size (FSC length signals) and form (SSC signals) to eliminate false-positive results (data not shown). Subsequently, gates were fixed around these three clusters for classification.

As dead cells were obtained through harsh microwave treatment, partial cell disintegration was observed. Consequently, the measured cell count in the cell suspension is dependent on viability of the biomass and different from the volumetric mixing ratio. This was considered in the target ratio of viable and dead cells which is given in Table 2 as “expected ratio,” calculated via Eqs. 1–4: results of each measurement series are given, comprising PBS buffer containing few particles, unfiltered SSL medium, and filtered SSL medium as background containing large amounts of particles.

**Table 2:** Overview on calibration procedure for flow cytometry-based method development. Each volumetric mixing ratio of viable (V) and dead (D) cell suspensions is listed with expected target ratios which are compared to measured values for cells in PBS buffer, filtered SSL medium, and unfiltered SSL medium. All measurements were carried out in technical triplicates, the measurements with PBS buffer were additionally carried out in duplicates

Volumetric mixing ratio of viable/dead cells		V/D (%)		V/D (%)		V/D (%)		V/D (%)		V/D (%)		V/D (%)		V/D (%)	
		100	0	80	20	60	40	50	50	40	60	20	80	0	100
PBS buffer pre-culture 1 (%)	Mean	99.7 ± 5.3	0.3 ± 0.2	99.5 ± 0.8	0.5 ± 2.2	83.8 ± 5.5	16.2 ± 8.5	69.2 ± 5.1	30.8 ± 1.2	56.6 ± 8.1	43.4 ± 5.2	35.4 ± 6.2	64.6 ± 9.1	0.0 ± 0	100.0 ± 4.7
Expected ratio (%)		100	0	89	11	75	25	66	34	57	43	33	67	0	100
PBS buffer pre-culture 2 (%)	Mean	100.0 ± 4.1	0.0 ± 0	91.2 ± 27.5	8.8 ± 3.1	80.3 ± 18.0	19.7 ± 5.9	76.4 ± 14.0	23.6 ± 4.2	72.7 ± 4.4	27.3 ± 1.6	50.7 ± 7.8	49.3 ± 1.6	0.0 ± 0	100.0 ± 31.5
Expected ratio (%)		100	0	93	7	84	16	77	23	70	30	46	54	0	100
Filtered SSL medium (%)	Mean	100.0 ± 4.9	0.0 ± 0.0	91.1 ± 3.2	8.9 ± 7.6	81.3 ± 8.3	18.7 ± 17.0	52.5 ± 2.3	47.5 ± 3.4	41.5 ± 2.7	58.5 ± 3.8	20.7 ± 2.8	79.3 ± 8.8	0.0 ± 0	100.0 ± 16.6
Expected ratio (%)		100	0	81	19	61	39	51	49	41	59	21	79	0	100
Unfiltered SSL medium (%)	Mean	100.0 ± 9.9	0.0 ± 0	81.3 ± 1.5	18.7 ± 1.1	61.4 ± 2.8	38.6 ± 2.1	53.6 ± 7.1	46.4 ± 4.8	42.7 ± 0.5	57.3 ± 1.1	21.0 ± 1.1	79.0 ± 0.9	0.0 ± 0	100.0 ± 22.1
Expected ratio (%)		100	0	80	20	60	40	50	50	40	60	20	80	0	100

**Fig. 2:**
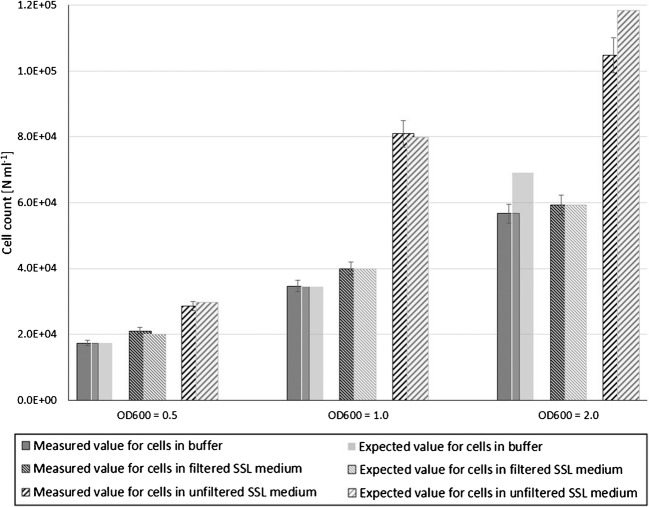
Impact of biomass concentration on yeast quantification. Comparison of measured and expected values for cells in buffer, filtered spent sulfite liquor, and unfiltered spent sulfite liquor. Variation of biomass was achieved by adjusting the optical density (OD_600_). Cell concentration is given in particles (*N*) per milliliter

**Fig. 3:**
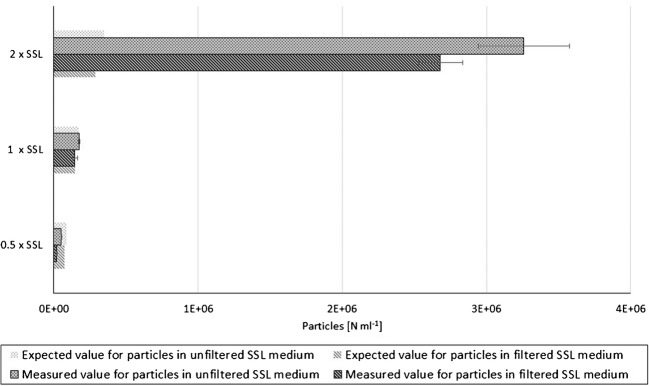
Impact of the amount of SSL particle background on spent sulfite liquor quantification. Comparison of measured and expected values for SSL in filtered and unfiltered medium. Concentration of SSL particles was varied by using the SSL media (1 × SSL), half (0.5 × SSL), and double (2 × SSL). Particles are given in particles (*N*) per milliliter

Figure 2 shows the impact of biomass concentration on the measurement at viable/dead mixtures of V40/D60 according to Table 1. At higher biomass concentrations, deviations between measured and expected values are clearly visible, dependent on the presence of high particle concentrations. This is further underlined by additional measurements at V50/D50, where the amount of spent sulfite liquor background was increased considerably. In such circumstances, the measurement capabilities of flow cell and detectors reach their limitations. The instrument software reduces data acquisition dependent on high particle concentrations to avoid data overload, which in turn leads to inaccurate results as illustrated in Fig. 3. This stresses the absolute necessity for proper sample dilution before measurement, as the method cannot cope with particle concentrations above 1 × 10^6^ particles mL^−1^. Subsequent measurements of regular samples were diluted accordingly.

**Fig. 4:**
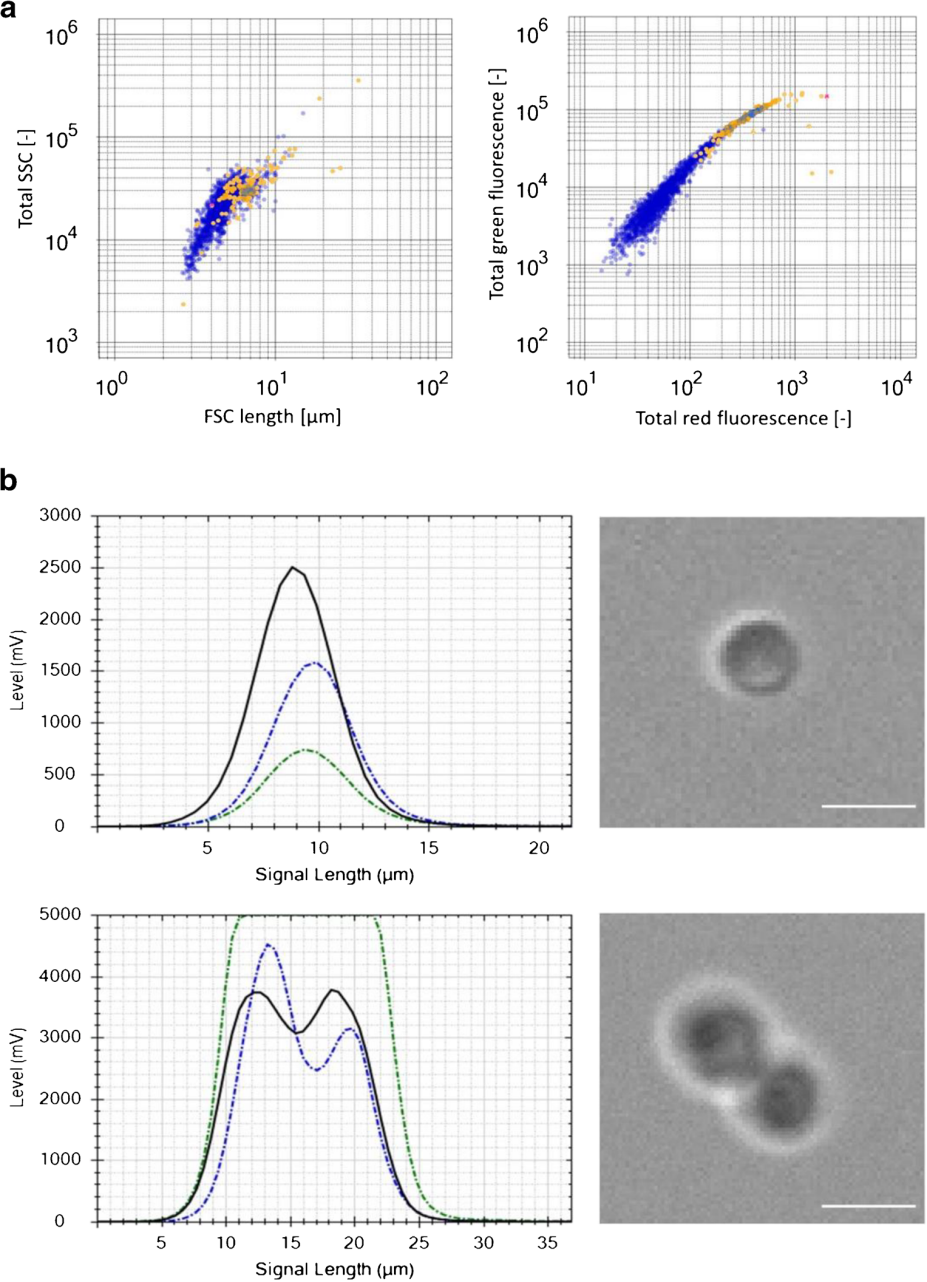
Morphological classification of viable yeast cells. **a** Classification according to sample length and total SSC signals to distinguish between single cells (blue) and budding cells (orange). **b** Signal shape profiles of single cells and budding cells: FSC signal (black), SSC signal (blue), and green fluorescence signal (green). Corresponding image-in-flow picture taken by the flow cytometer’s camera. White line signifies 10 μm

In order to further characterize biomass, signal curve properties of various detector signals can be used to differentiate morphological aspects. As explained by Dubelaar et al. [43], forward scatter (FSC) and sideward scatter (SSC) signals represent size, shape, and overall morphology of measured elements [43]. By using the flow cytometer, it was possible to distinguish between single cells and agglomerates featuring budding cells, thereby illuminating further physiological aspects. Morphological classification of single and budding cells is summarized in Fig. 4. Based on previously established morphological classes for yeast analysis [39], firstly, all viable yeast cells were detected and, secondly, discrimination between single or budding yeast cells was possible (Fig. 4a). Signal shape profiles of single and budding cells with corresponding images taken by the camera of the flow cytometer are shown in Fig. 4b. Due to high florescence stemming from budding cell agglomerations, saturation of green fluorescence signals can be observed. This is dependent upon detector sensitivity settings and cannot be wholly avoided if a wide range of particle sizes needs to be covered in a single measurement.

### Verification of biomass quantification against low and high particle backgrounds in a chemostat process

Upon successful establishment, the method was tested on its applicability in continuous cultivation. For this purpose, a chemostat experiment with unfiltered and filtered spent sulfite liquor with minimal nutrient supplementation was performed (see the “Spent sulfite liquor (SSL) medium” section). Using this approach, different biomass concentrations could be studied by flow cytometry under continuous conditions as the insufficient supply of media components resulted in a gradual wash out of cells after the initial YPD medium batch phase.

**Fig. 5:**
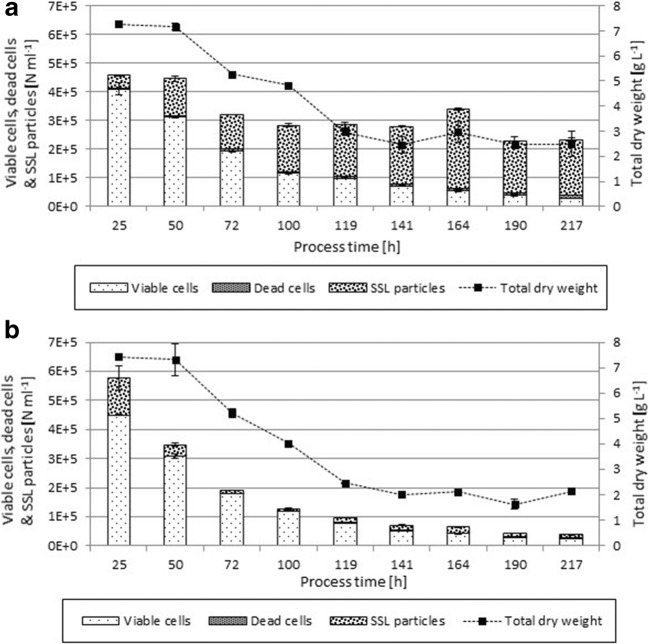
Application of flow cytometry method in a chemostat process. Particle populations across process time including total dry weight (g L^−1^), viable cells, dead cells, and SSL particle background for unfiltered (**a**) and filtered (**b**) SSL medium. Particle and cell concentration are given in particles (*N*) per milliliter

Figure 5 displays the concentration of viable cells and dead cells as well as the particle content of unfiltered (Fig. 5a) or filtered (Fig. 5b) SSL medium in continuous chemostat cultivations. The gravimetrically determined dry weight declined after the batch phase, reaching a steady state proportional to the total cell count of viable and dead cells measured in flow cytometry (Fig. 5). The decline visualizes the wash out of the cells by the constant feed and harvest rate. Wash out also led to a consistent low count of dead cells. The spent sulfite liquor particle concentration for unfiltered (Fig. 5a) or filtered (Fig. 5b) SSL medium was also reaching a steady value. Due to constant feeding of SSL medium, the relatively particle-free YPD batch medium was replaced. The count of spent sulfite liquor particles eventually reached the value present in the respective SSL feed medium.

The concentration of particles in unfiltered SSL medium are up to 20 times higher compared to filtered SSL medium, which nicely illustrates the effect of the pre-filtration procedure. Regarding viability in unfiltered and filtered chemostat cultivations, no effect of the different spent sulfite liquor particle content can be found.

To summarize, the method enables quantification of viable and non-viable cell populations against high particle backgrounds in chemostat experiments. Additional information is obtained through quantification of said particle backgrounds. Thereby, the process can be assessed in ways that are not possible through common monitoring of total dry weight.

### Monitoring of a retentostat process with accumulation of particle background

For process design of a continuous cultivation with cell retention, the physiology of the cells is essential. For that reason, the flow cytometry method was used as monitoring tool targeting physiological assessment over time during spent sulfite liquor fermentation in a retentostat process. Employing cell retention has the advantage of uncoupling yeast growth from product formation. That way, higher feed rates can be used and less substrate is needed for continuous formation of biomass [44, 45]. On the other hand, the use of membrane systems for bioprocessing of spent sulfite liquor represents a significant challenge in terms of biomass monitoring: while the particle background of a chemostat in steady state is equal to the particle background of the feed medium, the particle background in a cell retention process leads to accumulation of dirt particles over time.

This process was specifically designed for ethanol production and therefore divided into biomass accumulation phases under aerobic conditions (I batch and II retentostat) followed by an anaerobic, catalytically active phase (III retentostat) for production of ethanol from minimal spent sulfite liquor medium.

**Fig. 6:**
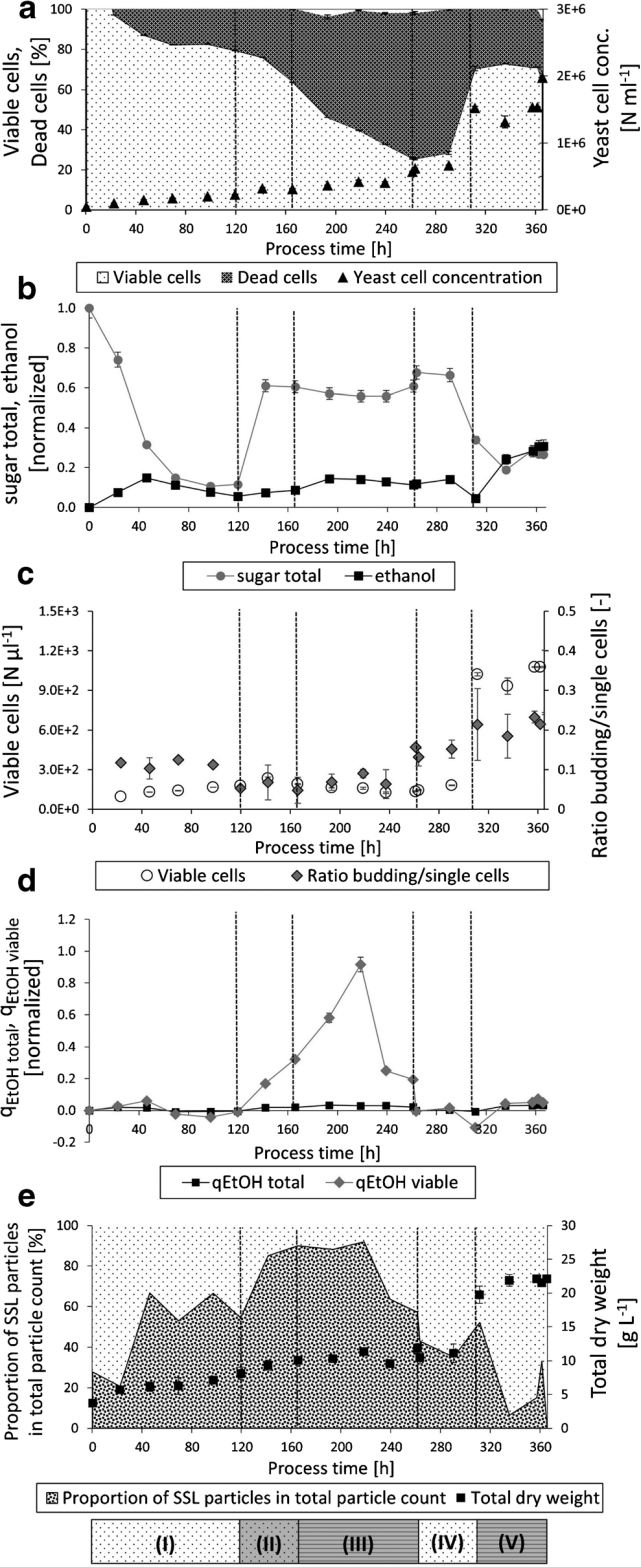
Monitoring of cell physiology and particle background of spent sulfite liquor fermentation in a retentostat process. Dotted lines distinguish process phases (I)—(V): I: 0–120 h/II: 120–166 h/III: 166–261 h/IV: 261–312 h/V: 312 h–end. **a** Ratio between viable and dead cells according to total particle count in %, yeast cell concentration in cells (*N*) per milliliter. **b** Ethanol titer and total residual sugar concentration (normalized values). **c** Viable cells in *N* per microliter, ratio between number of single and budding cells as an indicator for physiological budding activity. **d** Specific ethanol productivity *q*_Ethanol_ calculated via total dry weight and via viable cell dry weight (normalized values). **e** Ratio of spent sulfite liquor particles against total particle count in %, total dry weight in grams per liter

The assessment of viability provided valuable insight: phases I and II displayed a steady decrease of viability; during phase III a massive drop of viability (see Fig. 6a) was registered by the flow cytometry method. Additionally, the residual sugar concentration was consistently high, with a corresponding low ethanol titer (see Fig. 6b). To promote cell growth and increase viability, essential nutrients were pulsed to the reactor and conditions were switched to aerobic batch mode (IV, 266 h). Using the flow cytometry-based method, an increase in viability could successfully be detected during this second batch phase (IV) (Fig. 6a). Moreover, a higher of biocatalyst, i.e., viable biomass in the reactor, led to increased sugar uptake and ethanol titers.

Furthermore, morphological assessments shown in Fig. 6c demonstrate the increasing ratio of buddying cells to single cells at higher viability values. Consequently, when an improvement of overall viability and depletion of major sugars (see phase IV; Fig. 6b) could be observed, the process was switched back to ethanol production by anaerobic retentostat (phase V).

Quantification of viable biomass also enables a more thorough assessment of productivity. Specific ethanol productivity *q*_ethanol_ calculated from viable cells clearly shows a massive increase in phase III (see Fig. 6b) as opposed to *q*_ethanol_ calculated from total dry weight. This indicates that although overall viability declined, the population of viable cells displayed enhanced productivity.

In addition, the flow cytometry data demonstrate the characteristic problem of cell retention: holding back dead cells and SSL particles. This is illustrated in a twofold way: Fig. 6bX displays a high presence of dead cells in phase V at the end of the cell retention process. At the same time, Fig. 6e shows that between 280 and 360 h the percentage of SSL particle background decreased compared to the increasing cell concentration in the reactor (see Fig. 6e).

## Discussion

A novel method capable of identifying and quantifying the following particle populations in complex spent sulfite liquor medium was implemented: viable yeast cells, dead yeast cells, and media background containing solid particles. In addition, yeast cell morphology and physiology can be assessed.

### Advantages, disadvantages, and comparability of the method

In this study, flow cytometry was used to combine viability assessment and morphological analysis. Potential online use is possible but challenging as will be discussed in the “Applicability of the method” section. The method was specifically tailored to measurements in complex medium with high particle background. This signifies a fast and potent alternative to conventional offline measurements like dry cell weight and optical density which cannot distinguish between viable cells and media background. In addition, enhanced insight into yeast physiology is generated through simultaneous use of fluorescent viability staining and morphological assessment: information on overall viability, size distribution of media background and/or yeast cells can be obtained through one single measurement. Theoretically thousands of particles can be measured in a matter of minutes. Additionally, morphological cell features can be determined, even down to individual particles. This is especially useful to assess yeast physiology in distinct process stages through analysis of the budding division process. Other methods generally only provide an overview on viability and are time consuming [16, 46, 47]. The here-presented method could also be used with non-particle containing media. In such circumstances simpler biomass monitoring techniques would also be applicable, however information on non-viable biomass populations would be lost.

To establish the method, a comprehensive calibration procedure was used: mixtures of viable and dead cells in media containing different numbers of particle populations were measured. Table 2 (see the “Development of flow cytometry-based method” section) provides an overview on measurement errors dependent on biomass and media particle content. Naturally, samples containing high particle concentrations are challenging. However, standard deviations between actual and expected values were consistently below 10%.

However, a diverse particle population in the medium is challenging: to guarantee high information content across all process phases, adequate fluorescence detector sensitivity settings must be found for individual biomass and media combinations. In early process phases, detectors must be sensitive enough to detect viable biomass, and in later process stages, however, any signal saturation should to be avoided as it signifies a loss of information [33]. Furthermore, it should be noted that fluorescence spectral overlap might result in misleading signals. Depending on fluorescence intensity, green fluorescence can also be registered by the red fluorescence detector as a misleading artifact [48]. The flow cytometer analysis software CytoClus used in this study did not feature any fluorescence spectral overlap compensation. Also, deviations in the red fluorescence originating from particle interaction with PI could be observed. Consequently, biomass identification is not only based on fluorescence but also on size and form to eliminate false-positive results. The method cannot cope with unlimited particle concentrations. As a result, particle concentration in samples must be kept under 1 × 10^6^ particles mL^−1^ and verified in a preliminary measurement to avoid data overload and inaccurate results.

Disadvantages also include size-exclusion effects: small elements are generally over-represented due to the characteristics of the sampling tube (diameter 5 mm). However, such effects are hardy relevant when dealing with yeast due to its small size compared to other organisms like filamentous fungi.

### Applicability of the method

This method is a potent tool for at-line characterization of bioprocesses featuring complex media. If online applicability is implemented, the method can also be used for routine monitoring tasks. The use of commercialized live/dead cell viability assays is possible as well. However, its application is dependent on the wavelength of fluorescence emission of viability dyes and corresponding fluorescence detector specifications.

Potential online applicability would be possible in combination with automated sampling and sample processing. For this purpose, sampling, dilution, and addition of fluorescent dyes need to be performed in a modular process analytical (PAT) system with a connected flow cytometer [33]. However, it should be noted that the method is currently still used as an at-line method. For a robust use in process control, online applicability would have to be implemented first.

The developed method can shed a light on the complex bioprocessing of spent sulfite liquor, which features a high solid particle presence which would interfere with measurement when using other techniques apart from flow cytometry. The application of the method in a simple chemostat process and a complex cell retention process gave significant deeper insight into the physiology of the yeast cells and on the accumulation of solid particles. The additional information can be used for process design, targeting the physiological optimization and thus the productivity and performance of the process. For instance, the assessment of specific productivity is much more accurate when the actual value for viable biomass is known.

The main distinguishing feature of the here-presented flow cytometry method is its robustness and high information gain despite complex media backgrounds. In addition, viable and dead cell populations can be clearly distinguished using flow cytometry.
